# Scientific connotation of the compatibility of traditional Chinese medicine from the perspective of the intestinal flora

**DOI:** 10.3389/fphar.2023.1152858

**Published:** 2023-07-19

**Authors:** Yuan Gao, Xiaoxia Wu, Ning Zhao, Dong Bai

**Affiliations:** ^1^ Fang Zheng Center, Institute of Basic Theory for Chinese Medicine, China Academy of Chinese Medical Sciences, Beijing, China; ^2^ Experimental Research Center, China Academy of Chinese Medical Sciences, Beijing, China; ^3^ Department of Pharmacy, Xiyuan Hospital, China Academy of Chinese Medical Sciences, Beijing, China

**Keywords:** Chinese medicine, traditional Chinese medicine, compatibility, synergy and attenuation, contraindicated combination, intestinal flora

## Abstract

Revealing the connotation of the compatibility of Chinese medicines (CM) is a requirement for the modernization of traditional Chinese medicine (TCM). However, no consensus exists on the specific mechanism of traditional Chinese medicine compatibility (TCMC). Many studies have shown that the occurrence and development of diseases and the efficacy of CM are closely related to intestinal flora (IF), which may provide a new perspective to understand the theory of TCM. This study aimed to summarize the relationship between the changes in IF before and after the compatibility of different drugs and the synergistic, toxicity reduction, and incompatibility effects of drug pairs from the perspective of the effects of CM on the IF and the regulation of microbial metabolites. These studies showed that the effect of drug pairs on the composition of the IF is not a simple superposition of two single drugs, and that the drug pairs also play a specific role in regulating the production of intestinal bacterial metabolites; therefore, it has a different pharmacodynamic effect, which may provide a perspective to clarify the compatibility mechanism. However, research on the interpretation of the scientific connotations of TCMC from the perspective of the IF is still in its infancy and has limitations. Therefore, this study aimed to summarize previous research experience and proposed to conduct a deep and systematic study from the perspective of drug pair dismantling, IF, intestinal bacteria metabolite, organism, and disease to provide a reference for scientific research on the compatibility mechanism of CM.

## 1 Introduction

Many Chinese medicines (CM) are effective but toxic to humans. By combining different CM, adjusting the bias, restraining the toxicity, and taking advantage of the strength of the efficacy of the CM, toxicity can be reduced and effectiveness can be increased. This combination has been widely recognized by ancient and modern physicians and is a feature of the clinical application of traditional Chinese medicine (TCM). In the compatibility theory of TCM, the mutual reactions between drugs can be summarized into seven situations, named “Qi Qing,” including “Dan Xing,” “Xiang Xu,” “Xiang Shi,” “Xiang Wei,” “Xiang Sha,” “Xiang Wu,” and “Xiang Fan.” Disclosing the connotation of the TCMC is required to modernize TCM; however, no consensus exists on the specific mechanism of traditional Chinese medicine compatibility (TCMC). The inability to scientifically clarify the connotations of compatibility has somewhat limited the development of TCM.

Intestinal flora (IF) has become a popular research topic in recent years. Imbalance of the IF is not only related to intestinal diseases, but also to hepatic, cardiovascular, and neurological diseases through the intestine-liver, intestine-heart, and intestine-brain axis (Lin Z. et al., 2016; Sampson et al., 2016). With the popularization of gene sequencing technology, several studies have shown that CM can promote beneficial bacteria, inhibit harmful bacteria, regulate the metabolites of bacteria, such as bile acids (Bas) and short-chain fatty acids (SCFAs), and thus exert a regulatory effect on the organism (Xu et al., 2017).

The IF is a key link between efficacy and CM. TCM theory states that a healthy human body needs not only to maintain harmony and unity with the external environment but also to maintain the balance of the internal environment. Maintaining the stability of the IF conforms to the concept of “holism” of TCM, and the IF can provide a new perspective to understand the TCM theory. From the perspective of the effect of CM on the IF and the regulation of microbial metabolites, this study summarized the relationship between the changes in the IF and the effects of synergism and toxicity reduction after CM combination, with a view for providing ideas for future systematic studies of the mechanism of CM compatibility.

## 2 Current situation of research on the relationship between the synergism effect of TCMC and IF

The principle of “Qi Qing” in TCM achieves the result of compatibility and synergy. “Xiang Xu” refers to a combination of drugs with similar performance, which have synergistic effects and enhance the original efficacy. “Xiang Shi” refers to a combination of drugs that have certain similarities in performance and efficacy, with one drug as the main drug and one as a supplement to improve the efficacy of the main drug. Both “Xiang Xu” and “Xiang Shi” can play a synergistic role. We believe that the increase in therapeutic efficacy is related to the specific regulation of the IF by the combination of the two drugs. The combination of two drugs may have a stronger regulatory effect on a certain bacterium, resulting in one plus one being greater than two, or it may be that the combination has a specific regulatory effect on a new bacterium and a different effect from that of a single drug. This may be one of the ways to clarify the synergistic effect of compatibility.

**FIGURE 1:**
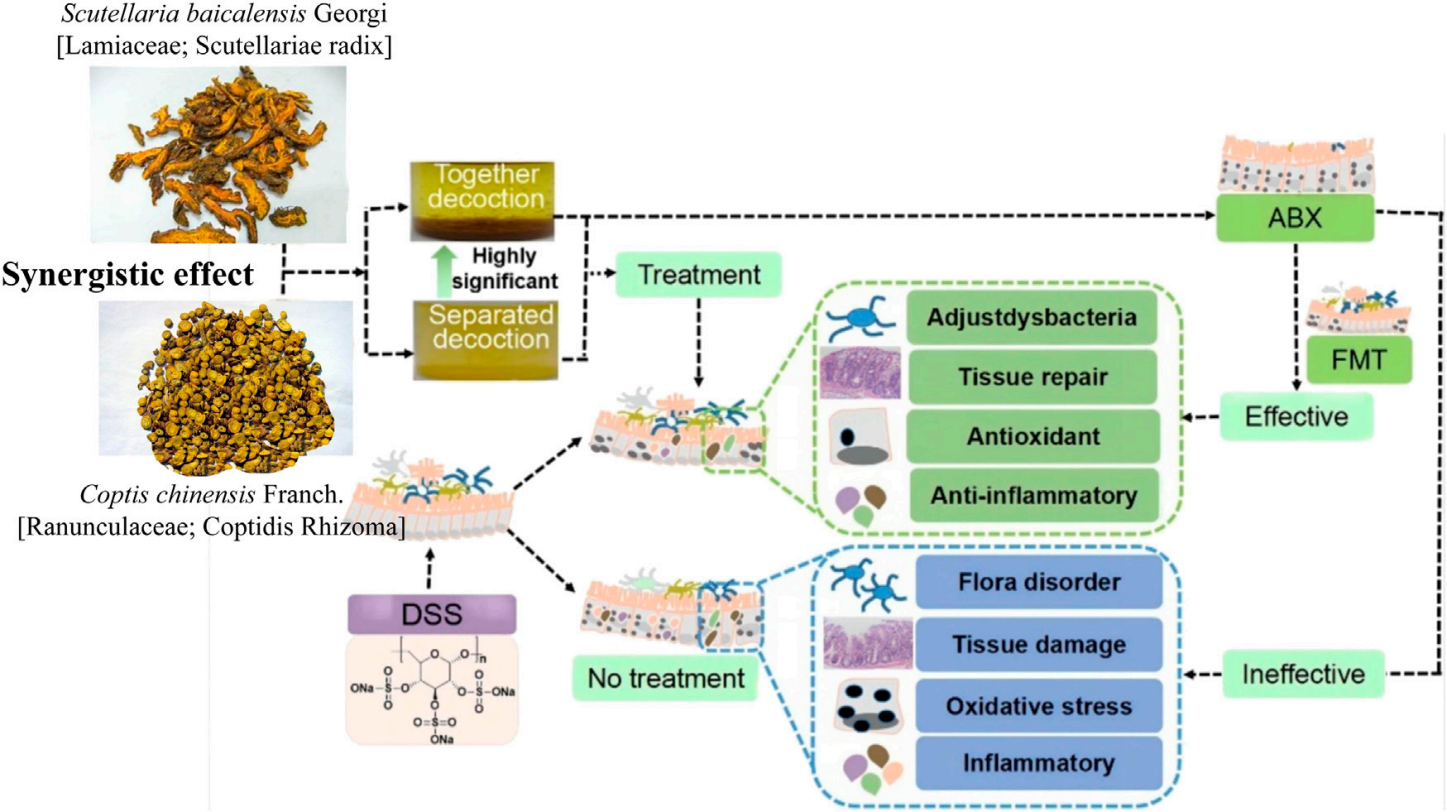
Therapeutic effect of the combination of *S. baicalensis* and *C. chinensis* on DSS-induced ulcerative colitis in mice by acting on intestinal flora (DSS, dextran sulfate sodium; ABX: Antibiotic interference, FMT: Colony transplantation) (Liu et al., 2022).

*Scutellaria baicalensis* Georgi (Lamiaceae; Scutellaria radix) (*Scutellaria baicalensis*)- *Coptis chinensis* Franch (Ranunculaceae; Coptidis Rhizoma) (*C. chinensis*) compose a classical “drug pair” applied in clinical practice to dispel heat, dryness, and dampness. Hyperglycemia, dyslipidemia, inflammation, and insulin resistance in type 2 diabetes mellitus (T2DM) were ameliorated after oral administration of *S. baicalensis* and *C. chinensis*, particularly the combined extract. Moreover, the effects of the combined extracts were more remarkable than those of the single-drug treatment (Cui et al., 2018). The unique efficacy of *S. baicalensis*—*C. chinensis* may be related to the regulation of glucose and lipid metabolism and improvement of the IF (Ding et al., 2019). *In vitro* experiments showed that single or combined use of *S. baicalensis* and *C. chinensis* can promote the growth of beneficial bacteria *Bifidobacteria* and *Lactobacilli* in the intestinal tract of normal and T2DM model rats and inhibit the growth of harmful bacteria *Enterococcus* and *Enterobacter*; and the effect of drug pairs is stronger than that of a single drug (Xu, 2014). Acidic metabolites of beneficial intestinal bacteria, such as *Bifidobacteria* and *Lactobacilli*, can reduce the local pH of the intestine and produce substances with broad-spectrum antibacterial effects, thereby improving intestinal function by inhibiting the growth of intestinal and conditional pathogens. This indicates that the combination of *S. baicalensis* and *C. chinensis* can have a positive effect on IF. Liu (Liu et al., 2022) studied the effects of separate and combined applications of *S. baicalensis* and *C. chinensis* on ulcerative colitis (UC) induced by the administration of dextran sulfate sodium (DSS) in mice, as shown in Figure 1. These results revealed that the combined application of *S. baicalensis* and *C. chinensis* significantly relieved colon inflammation in mice. Notably, the protective effects of *S. baicalensis* and *C. chinensis* against colon inflammation were weakened when the antibiotic mixture was partially consumed by the gut microbiota. A fecal microbial transplantation experiment further proved that the therapeutic effects of *S. baicalensis* and *C. chinensis* on UC were closely related to IF. The results of 16S rRNA sequencing showed that the group treated with combined applications of *S. baicalensis* and *C. chinensis* exhibited a higher intestinal microbial diversity and the IF composition than those of the separate groups; the abundance of *norman_f_Muribaculateae* increased relatively, and the abundance of *Bacteroides*, *Akkermania* and *Lactobacillus* also changed, but the difference was not significant. Correlation analysis showed that the bacterial flora regulated by *S. baicalensis* and *C. chinensis* was closely related to inflammatory factors in UC treatment. These results indicate that the therapeutic effect of the combination of *S. baicalensis* and *C. chinensis* is better than that of a single drug, which is related to the regulation of the IF and inhibition of inflammation.

*S. baicalensis* and *Sophora japonica* Linn (Fabaceae; Sophora Flos) (*S. japonica*) were originally obtained from Renzhai Zhizhi and are clinically applicable to hypertensive patients with hyperactive liver fires. Guan (Guan et al., 2021) established spontaneously hypertensive models to explore the renal protective effects of a combination of *S. baicalensis* and *S. japonica* against chronic kidney disease. The results showed that the combination of *S. baicalensis* and *S. japonica* significantly ameliorated the severity of renal injury induced by hypertension compared with effectiveness of single drugs. The antihypertensive effect and renal protection of *S. baicalensis* and *S. japonica* are affected after the bacterial flora is disturbed by antibiotics, which indicates that the combination of *S. baicalensis* and *S. japonica* plays a therapeutic role by acting on IF. The regulation of the intestinal microecological balance may be a mechanism of action of *S. baicalensis* and *S. japonica* in the treatment of hypertension and renal damage. The regulatory effect of the combination of *S. baicalensis* and *S. japonica* on the IF was different from that of the single drugs. Compared with the model group, the diversity of the IF in the combination group increased, and the ratio of *Firmicutes*/*Bacteroidetes* (F/B) decreased. Compared with model group, the relative abundances of *Prevotella-9* and *Akkermansia* were higher in the *S. baicalensis* group, whereas those of *Corynebacterium* and *Prevotella-9* were increased in the *S. japonica* group. The relative abundance of *Lactobacillus* increased, and that of *Clostridiales* decreased in the *S. baicalensis* and *S. japonica* group. *Prevotella-9*, *Lactobacillaceae*, and *Bifidobacteriaceae* are beneficial bacteria. *Lactobacillus* can reduce the serum cholesterol level of hyperlipidemic rat models by improving the balance between intestinal microorganisms and increasing the intestinal transit time (Xie et al., 2011), which is closely associated with metabolic diseases. *Clostridiaceae*, an indole-positive bacterium, is positively correlated with indole, which has negative effects on the kidney (Niwa, 2013). With an increase in the abundance of dominant bacteria, the intestinal barrier improves, and the change in dominant bacteria reduces indole accumulation, further inhibiting oxidative stress activation in the kidneys. Olfr78 regulates renin secretion and increases blood pressure. Activated GPR41 relaxes blood vessels and lowers blood pressure. *S. baicalensis* and *S. japonica* increased SCFA production, inhibited the release of inflammatory factors, and regulated blood pressure by decreasing the expression of Olfr78 and increasing GPR41 expression, thereby alleviating kidney damage. These results indicate that the hypotensive effects of *S. baicalensis* and *S. japonica* in rats may be related to the regulation of IF, thereby increasing SCFA levels (Pluznick, 2014).

Gegen Qinlian Decoction (GQD), derived from the Treatise on Febrile Diseases, is a typical prescription for the clinical treatment of acute enteritis which is composed of *Pueraria montana* var. *Lobata* (Willdenow) Maesen and S. M. Almeida ex Sanjappa & Predeep [Fabaceae; Pueraria Lobata Radix] (*P. montana*), *S. baicalensis*, *C. chinensis*, and *Glycyrrhiza uralensis* Fisch (Fabaceae; Glycyrrhizae radix et rhizoma) (*G. uralensis*). The study found that GQD can restore the diversity of IF and significantly increase the relative abundance of bacteria that generate SCFAs, thus increasing the concentrations of acetic acid, propionic acid, and butyric acid in feces. Increased SCFAs can inhibit the HADC and NF-κB pathways to alleviate inflammatory reactions in the intestinal mucosa. GQD treatment of diarrhea may modulate the gut microbiota and increase SCFA levels (Liu et al., 2019). Chen (Chen et al., 2020) found that GQD and its different compatibilities had different therapeutic effects on acute enteritis, and GQD and the whole prescription without *G. uralensis* had more obvious anti-inflammatory and mucosal reconstruction and ulcer repair effects on colon tissue. Based on this difference, Chen analyzed the diversity of IF. Alpha and beta diversity showed that the IF composition in each group was significantly different. Compared with the model group, GQD and its different compatibilities significantly reduced the relative abundance of *Clostridium_sensu_stricto_1* which is associated with intestinal inflammatory diseases. It can be seen from the results of the group of GQD without *S. baicalensis* and *C. chinensis* that the combination of *P. montana* and *G. uralensis* increases the abundance of *Bacteroidales_S24-7_ukn*, which is a beneficial bacterium, and *Allobaculum*, which is an SCFA-producing bacterium, while the abundance of pathogenic bacteria *Parabacteroides* decreases, but at the same time, the abundance of *Desulfovibrio*, which is toxic to colon cells, increases. The genomes of *Bacteroidales_S24-7_ukn* and *Akkermania* both encode the ability to produce propionate, and the increase of propionate is closely related to the stability of intestinal inflammation (Borton et al., 2017); *Allobaculum* can rapidly ferment glucose to produce lactic acid and butyric acid; *Parabacteroides*, as a pathogen in infectious diseases, can induce inflammation and immune disorder (Larsen, 2017); *Desulfovibrio* can damage the intestinal barrier by producing lipopolysaccharides (Beerens and Romond, 1977). These results indicated that the combination of *P. montana* and *G. uralensis* can inhibit the occurrence of inflammation and metabolic disorders. The results showed that the combination of *S. baicalensis* and *C. chinensis* increased the relative abundance of the beneficial bacterium *Akkermannia* and decreased that of the pathogenic bacterium *Parabacteroides*, indicating that the combination of *S. baicalensis* and *C. chinensis* plays an important role in regulating IF, and this compatibility could play a positive role in acute enteritis. Simultaneously, *Allobaculum* abundance decreased in the *S. baicalensis* and *C. chinensis* group. Combined with the results of the GQD group, the GQD without the *G. uralensis* group and the GQD without *P. montana*, shows that the compatibility of *G. uralensis* and *P. montana* also plays a key role in the regulation of IF. Therefore, it was concluded that *S. baicalensis* and *C. chinensis* are the key components in GQD that regulate the balance of IF, and the compatibility of *G. uralensis* and *P. montana* enhances the regulation of IF. It can be found that there is a complex network relationship between disease, flora and drugs. The differences at the gene level between different administration groups and model groups may be the biological basis for the different compatibilities of GQD to produce different effects.

Banxia Xiexin Decoction (BXD), derived from the Treatise on Febrile Diseases, is widely used to treat digestive system diseases, such as gastritis, enteritis, gastric ulcer, and gastrointestinal dysfunction. The whole prescription can be divided into the “Xinkai” compatibility unit of the combination of *Pinellia ternata* (Thunb.) Ten. ex Breitenb (Araceae; Pinelliae rhizoma) (*P. ternata*) and *Zingiber officinale* Roscoe (Zingiberaceae; Zingiberis rhizoma) (*Z. officinale*), the “Kujiang” compatibility unit of the combination of *S. baicalensis* and *C. chinensis*,, and the “Ganbu” compatibility unit of the combination of *Panax ginseng* C. A. Mey (Araliaceae; Ginseng radix et rhizoma) (*P. ginseng*), *Ziziphus jujuba* Mill (Rhamnaceae; Jujube fructus) (*Z. jujuba*), and *G. uralensis*. Previous studies have shown that BXD can reduce intestinal inflammation and treat ulcerative colitis by improving IF imbalance (Chen et al., 2021). Studies have shown that the coordination between IF, tight junction proteins, and the intestinal mucosal barrier plays an important role in maintaining the steady state of the intestinal barrier (Fan et al., 2019). Therefore, Zhang (Zhang et al., 2021; Dai et al., 2022) believed that antibiotic exposure leads to IF disorder in young rats, thus damaging the intestinal mucosal barrier, and that BXD and different disassembled prescriptions can regulate the IF structure, protect the intestinal mucosal barrier from pathological damage caused by antibiotic exposure, and improve the immune response. After antibiotic interference, the IF of young rats changed significantly. After treatment, the difference in the IF between the BXD group and blank group was significantly reduced, and the recovery effect of the BXD group was the best. By studying the flora composition at the genus level, it can be found that, compared with the model group, the BXD group and the different disassembled formula groups can significantly reverse the increase of *Klebsiella* and *Enterobacter* abundance caused by modeling, and the effect of “Xinkai” group is the most significant. At the same time, the abundance of *Bacteroides* and *Lactobacillus* increased in each treatment group, and the increase in *Lactobacillus* abundance in the BXD group was the most significant. The abundance of *Bacteroides* in “Xinkai” group and “Ganbu” group was the highest. *Enterobacter* is a common pathogenic bacterium that can be colonized by host inflammatory reactions to further increase the severity of intestinal inflammation (Li et al., 2020). *Klebsiella* is a conditional pathogen that causes respiratory and digestive tract infections (Li J. et al., 2019). *Bacteroides* play important roles in intestinal mucosal angiogenesis, intestinal microecological balance, and host immunity. *Lactobacillus* has beneficial effects on intestinal inflammation, oxidative stress, and symbiosis of microbiota (El-Baz et al., 2020). In summary, BXD and different decoctions can adjust the IF structure of antibiotic-exposed young rats. Among them, the “Ganbu” and “Xinkai” decoctions play a central role. The “Xinkai” group can effectively reduce the abundance of pathogenic bacteria, and has more advantages in regulating the balance of flora, while the “Ganbu” group can effectively increase the abundance of probiotics. Liang (Liang et al., 2021) studied the effect of BXD and its compatibility with gastrointestinal bacteria using *in vitro* antibacterial and bacteriostatic activity tests. *Helicobacter pylori* infection is closely associated with chronic gastritis and gastric mucosal damage. The research results show that the whole formula group has good bacteriostatic and bactericidal effects on *H. pylori*, followed by “Kujiang” group. The BXD and different compatibilities also have inhibitory effects on two harmful intestinal bacteria, *Escherichia cloacae* and *Enterococcus faecalis*, to varying degrees and are dose-dependent within a certain concentration range. The antibacterial effect of the BXD group and “Kujiang” group is the strongest. Therefore, it was speculated that the material basis of BXD against harmful bacteria is mainly composed of *Z. officinale*, *S. baicalensis*, and *C. chinensis*. When observing the effect of BXD on beneficial bacteria, it was found that the growth of beneficial bacteria was inhibited in “Kujiang” group, while the growth of *Bifidobacteria adolescentis* and *Lactobacillus acidophilus* was promoted in the whole recipe group, “Ganbu” group and “Xinkai” group within a certain concentration range. Thus, it is speculated that the “Kujiang” group in BXD can effectively inhibit the growth of pathogenic bacteria *in vitro*, while the “Ganbu” group can promote the proliferation of beneficial bacteria.

Furthermore, many studies have reported on the relationship between the synergistic effects of TCMC and IF, as shown in Table 1.

**TABLE 1:** Some research about the relationship between the synergism effect of TCMC and IF.

Drug pair	Model	Flora change	Action mechanism of drug pair
*S. baicalensis* and *C. chinensis* Xiao et al. (2020)	T2DM rats	The abundance of SCFAs-producing bacteria, such as *Parasutterella*, *Ruminiclostridium*, and *Ruminiclostridium 9*, increased significantly in the single drug and combination groups, while the abundance of *Escherichia* and *Shigella*, which produced secondary BAs, decreased	The combination of *S. baicalensis* and *C. chinensis* can effectively reduce the level of secondary BAs and increase the level of SCFAs, thus improving the metabolic spectrum disorder of T2DM rats
*C. chinensis* and *Z. officinale* Wei et al. (2022)	UC in mice	The abundance of *Bacillibacterium* increased in the berberine, 6-gingerol, and combination groups, while the abundance of *Verrucomicrobia* decreased significantly. The increase of *Akkermania muciniphila* destroys the intestinal mucosal barrier and aggravates intestinal inflammation. *Akkermania muciniphila* disappeared in the 6-gingerol and combination groups	The combination of berberine in *C. chinensis* and 6-shogaols in *Z. officinale* can inhibit intestinal inflammation, reduce macrophage infiltration and activation, reduce intestinal damage, reduce intestinal permeability, and improve intestinal microecology balance. The combination of berberine and 6-shogaols has a significant synergistic effect
*Artemisia caruifolia* Buch.-Ham. ex Roxb. [Asteraceae; Artemisiae annuae herba] (*A. caruifolia*) and *Pelodiscus sinensis* [Trionychidae; Trionycis carapace] (*P. sinensis*)Lin et al. (2021)	MRL/lpr lupus mice	*Lactobacillus, Allobaculum, Sutterella, Dehalobacillus, Coprococcus, Dorea, Oscillospira, and Desulfovibrio* are the bacterial groups specially regulated by *A. caruifolia*; *Ruminococcus* is a bacteria group specifically regulated by *P. sinensis*, and *Bacteroides, Parabacteroides,* and *Coprobacillus* are bacteria groups specifically regulated by the combination of *A. caruifolia* and *P. sinensis*	*A. caruifolia* and *P. sinensis* can improve the condition of lupus mice, and the treatment effect of *A. caruifolia* and *P. sinensis* is better, which may be related to the drug pair increases the abundance of bacteria negatively correlated with the disease
*Rheum palmatum* L. [Polygonaceae; Rhein radix et rhizoma] (*R. palmatum*) and *Astragalus membranaceus* var. *mongholicus* (Bunge)P.K.Hsiao [Fabaceae; Astragali radix] (*A. membranaceus*) Zhong, (2017)	Chronic renal failure rats	The compatibility of *R. palmatum* and *A. membranaceus* in different proportions can inhibit the reproduction of harmful bacteria *Escherichia coli* and *Enterococcus*, promote the growth of beneficial bacteria *Lactobacillus* and *Bifidobacterium*, and restore intestinal function	*R. palmatum* with different proportions of *A. membranaceus* can significantly reduce the level of metabolic toxins, regulate IF, enhance intestinal mucosal barrier function, promote the recovery of intestinal function, and play a role in treating chronic renal failure through the intestinal renal axis
*P. ginseng* and *Atractylodes macrocephala* Koidz. [Asteraceae; Atractylodis Macrocephalic rhizoma] (*A. macrocephala*)Wang et al. (2019)	Chemotherapy diarrhea mice	*A. macrocephala* oil or *P. ginseng* saponins cannot effectively improve the changes in the flora structure caused by chemotherapy and even have adverse effects on *Blautia*, *Parabacteroides*, and *Lactobacillus*. The combination of drugs can eliminate the adverse effects of a single drug on the IF, restore the *F*/*B* ratio, and specifically reduce the abundance of harmful bacteria *Bacteroides, Ruminococcus, Anaerotruncus, and Desulfovibrio*	The combination of *A. macrocephala* oil and *P. ginseng* saponins can effectively improve diarrhea and related pathological changes in mice caused by chemotherapy, which has an important relationship with IF, as shown in Figure 2
*A. membranaceus* and *Codonopsis pilosula* (Franch.) Nannf. [Campanulaceae; Codonopsis radix] (*C. pilosula*)Tang et al. (2021)	Mice with acute colitis	The structure of the damaged IF was effectively restored by the combination of drugs. The level of *Bacteroidetes* increased, while the levels of *Firmicutes* and *Proteobacteria* decreased. *Proteobacteria* is a flora specifically regulated by the combination of the two drugs	The combination of *A. membranaceus* polysaccharide and *C. pilosula* polysaccharide can improve the symptoms of mice with acute colitis, and the effect is better than that of a single drug. Its synergistic effect is related to the adjustment of IF, activation of aromatic hydrocarbon receptors in colon tissues, and the increase of isovalerate and butyrate in feces (Figure 3)
*C. chinensis* and *Rehmannia glutinosa* (Gaert.) Libosch. ex Fisch. et Mey. [Orobanchaceae; Rehmanniae radix] (*R. glutinosa*) Han et al. (2016)	T2DM KKAY mice	The combination of berberine and stachyose, the effective ingredient in *C. chinensis* and *R. glutinosa*, can significantly promote the proliferation of *Lactobacillus* and *Bifidobacterium* in the intestinal tract of mice, and its effect is significantly better than that of the berberine group and stachyose group	The combination of berberine and stachyose, the effective component of *C. chinensis* and *R. glutinosa*, can significantly improve the disorder of glucose and lipid metabolism, and its effect is superior to that of a single component. Its mechanism may be related to promoting the proliferation of intestinal probiotics
Baidu Decoction (BHD)Ye, (2020)	Fever rats	*Gypsum Fibrous* (*G. Fibrous*) decoction, *G. Fibrous* and *Anemarrhena asphodeloides* Bunge [Asparagaceae; Anemarrhenae rhizoma] (*A. asphodeloides*) decoction and BHD can all callback the *Alpha-proteobasteria, Selenomonadales, Rhodospirillales, Akkermaniacae,* Burkholderiaceae*, Acidaminocacaceae, Lachnospirace-ae_NK4 A136_group* changes caused by modeling	BHD and its disassembled prescriptions can significantly reduce the body temperature of rats with fever, which may be related to the signal pathway of NF-κB, maintaining the balance of body fluid components, regulating the biodiversity of IF, and improving the disorder of amino acid metabolism and lipid metabolism in the body
Sishen Pill (SP) Liu, (2019)	Diarrheal irritable bowel syndrome in rats	Compared with the model group, the abundance of *Allobaculum* and *Proteobateria* in the SP group was reduced, and the abundance of *unclassified_k__norman*, *Clostridium_sensu_stricto_1*, *Turicibacter*, and *Romboutsia* increased significantly	SP may play a role in the treatment of diarrhea - type irritable bowel syndrome partly by regulating the structure of IF, and there is a synergistic effect between the various groups
Quyu Huatan Tongmai Fang (QHTF)Miao et al. (2022)	Hyperlipidemic golden hamster	QHTF can improve the IF structure, significantly reduce the F/B ratio, increase the abundance of Bacteroidaceae*,* Porphyromonadaceae*,* Rikenellaceae*,* and *Pasteurella*, and reduce the relative abundance of *Coriobacterium*. The regulating effect of the Huatan group on the flora is closer to that of the QHTF group	QHTF and its compatibility group can improve hyperlipidemia to different degrees, and its mechanism may be related to regulating the IF structure and improving intestinal microecology. The compound group has the most significant effect, followed by the Huatan group
LianPo Yin (LPY) Wang Q. et al. (2022)	Ex colony fermentation of human intestinal bacteria	The relative abundance of *Escherichia-Shigella, Enterococcus, Bacteroides*, and *Bifidobacterium* was significantly reduced in the *C. chinensis* and *Houpoea officinalis* (Rehder and E. H. Wilson) N. H. Xia and C. Y. Wu [Magnoliaceae; Magnolia officinalis cortex] (*H. officinalis*). Compared with the drug pair group, LPY significantly increased the relative abundance of *Enterococcus*, *Bacteroides*, and *Bifidobacterium*, and decreased the relative abundance of *Klebsiella*	*C. chinensis* and *H. officinalis* have an antibacterial effect, while LPY promotes the growth of beneficial bacteria and has a positive regulatory effect on the human IF.

**FIGURE 2:**
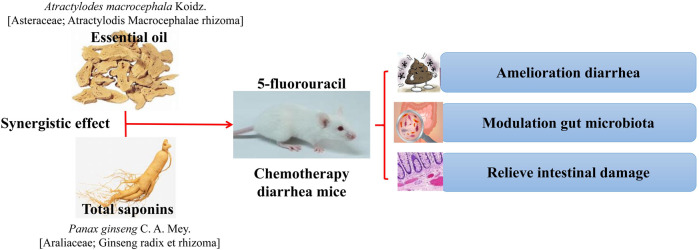
Combined use of *A. macrocephala* oil or *P. ginseng* saponins decreases chemotherapy-induced diarrhea in mice by affecting intestinal flora (Wang et al., 2019).

**FIGURE 3:**
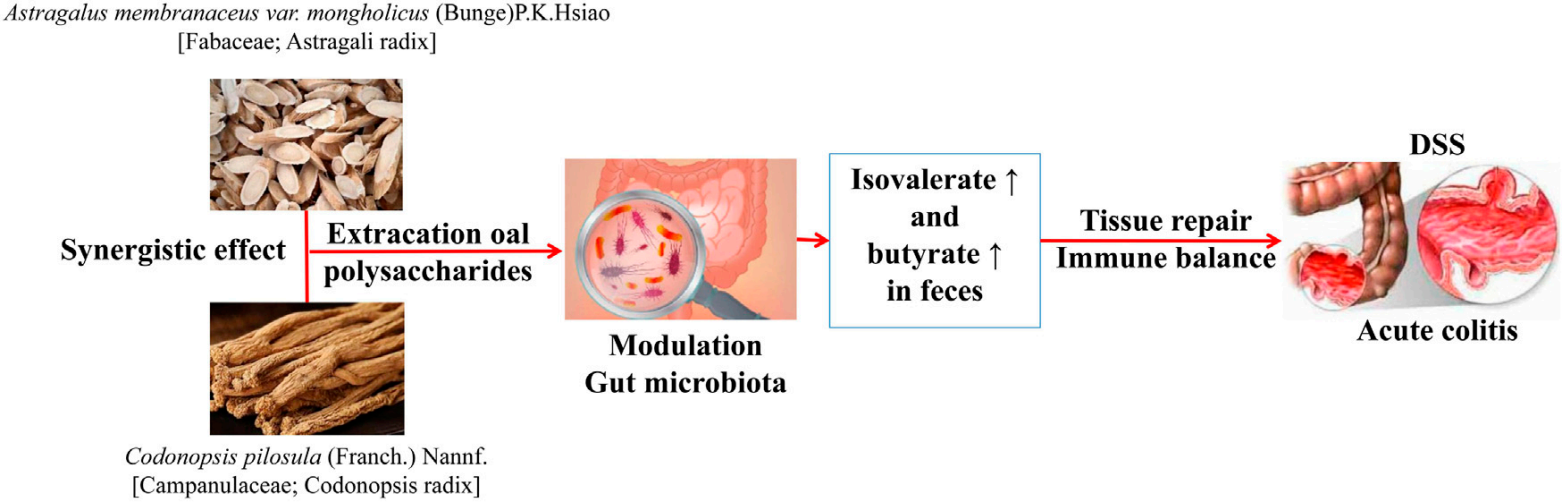
Therapeutic effect of *A. membranaceus* polysaccharide combined with *C. pilosula* polysaccharide on acute colitis mice by acting on intestinal flora (Tang et al., 2021).

## 3 Current situation of research on the relationship between the attenuation effect of TCMC and IF

Reasonable compatibility reduces drug toxicity and expands the scope of clinical applications. Although the mechanism of CM toxicity is very complex, current research shows that the IF is also an important factor affecting the toxicity of CM. The principle of “Xiang Wei” and “Xiang Sha” in “Qi Qing” achieves the result of toxicity reduction. “Xiang Wei” refers to the toxicity or side effects of one drug can be eliminated by another drug, and “Xiang Sha” refers to one drug can alleviate or eliminate the toxicity or side effects of another drug. “Xiang Wei” and “Xiang Sha” illustrate the same problem from two perspectives. We believe that the elimination or alleviation of toxic effects is related to the specific regulation of the IF by the combination of the two drugs. CM with toxicity or side effects may affect the structure of the IF, reduce the abundance of beneficial bacteria, and increase the abundance of harmful bacteria. After compatibility, the negative effects of CM with toxicity or side effects on the IF are eliminated, which has a positive effect on the body.

**FIGURE 4:**
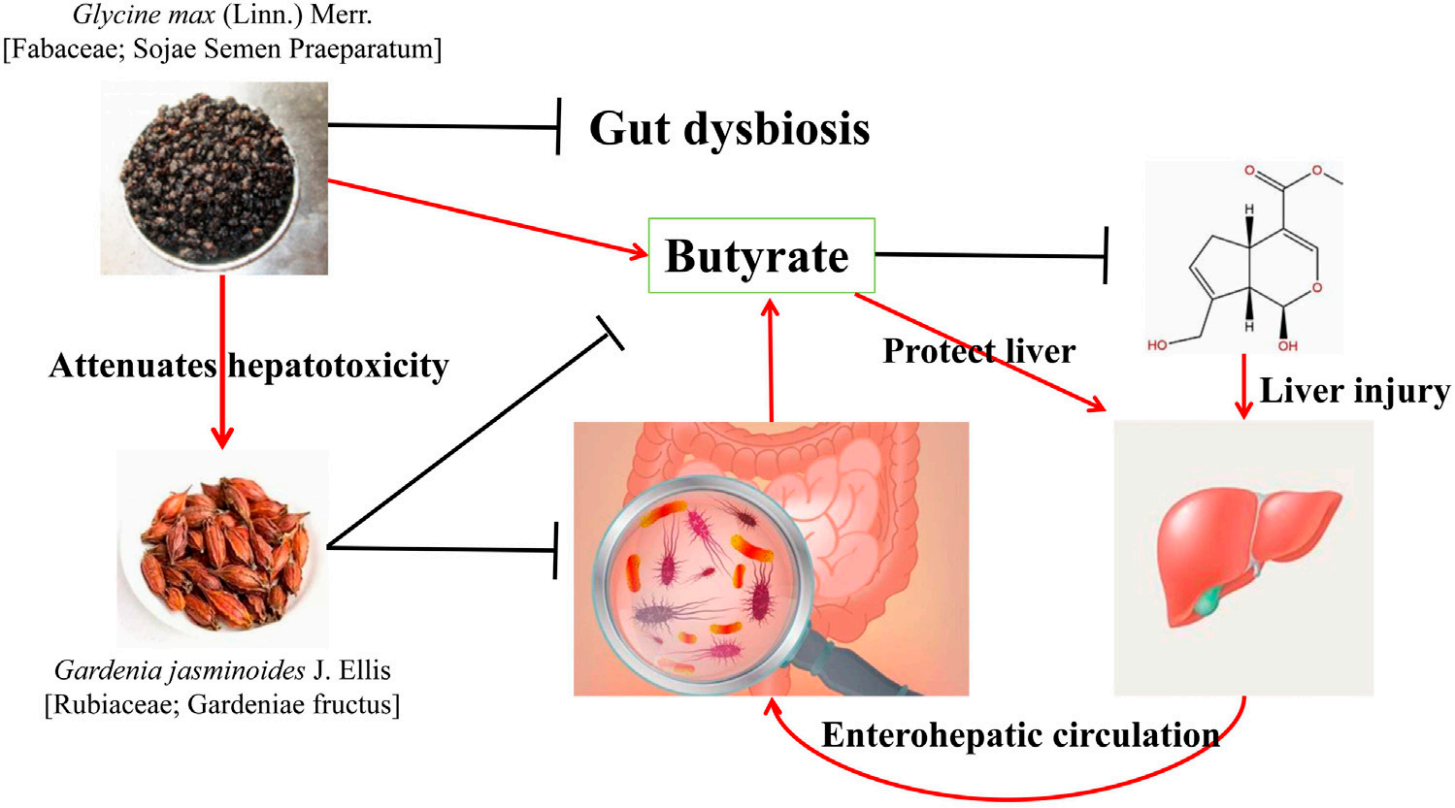
Combination of *G.* max and *G. jasminoides* reduces the toxicity of *G.* max by affecting the intestinal flora (Luo et al., 2021).

The combination of *Glycine* max (Linn.) Merr (Fabaceae; Sojae Semen Praeparatum) (*G. max*) and *Gardenia jasminoides* J. Ellis (Rubiaceae; Gardenia fructus) (*G. jasminoides*) is from the Zhizi Chi Decoction (ZCD) in Zhongjing Zhang’s Treatise on Febrile Diseases which is a classic prescription for treating insomnia caused by heat stagnation chest diaphragm (Shi et al., 2012). The combination of these two drugs reduced the liver toxicity of *G. jasminoides*. Luo (Luo et al., 2021) suggested that the improvement of *G.* max in *G. jasminoides* -induced liver injury was related to the IF. At the same dose, the hepatotoxicity of ZCD was significantly lower than that of the *G. jasminoides*. The IF analysis revealed that *G. jasminoides* affected the IF composition of mice, reduced the abundance of *Lactobacillus* and *Enterococcus*, and increased the abundance of *Parasutterella*. However, the abundance of the beneficial bacteria *Akkermania* and *Prevotella* increased significantly after *G. jasminoides* was combined with *G. max*. *Prevotella* can promote glycogen storage in the mouse liver and maintain glucose homeostasis in the host (Purushe et al., 2010). In addition, *G. jasminoides* reduced the level of butyrate in feces, which was reversed after combination with *G. max*. When the level of butyrate increases, it plays a protective role in the liver by improving the integrity of the colon and promoting the activation of Nrf2. The combination of *G.* max and *G. jasminoides* cured *G. jasminoides*—induced liver injury by regulating the microbiota and promoting butyrate production (Figure 4). Chen (Chen L. et al., 2019) found that ZCD can maintain the relative balance of the IF better than *G.* max or *G. jasminoides* can, via an *in vitro* study. Therefore, *G. jasminoides* has a negative impact on the IF, and the compatibility of *G.* max and *G. jasminoides* can not only benefit the IF but also positively reverse the disorder of the IF caused by *G. jasminoides*.

Realgar is a mineral and heavy-metal CM with significant therapeutic effects in the treatment of leukemia and various solid tumors. However, there are several adverse reactions, including intestinal, cardiac, and liver toxicities. The compatibility of Realgar and *S. miltiorrhiza* Bunge (Lamiaceae; Salvias miltiorrhizae radix et rhizoma) (*Salvia miltiorrhiza*) was derived from the Compound Huangdai Tablet, which was formulated by Professor Shilin Huang. Clinical practice has confirmed that the treatment for acute promyelocytic leukemia is effective, with a high cure rate and mild adverse reactions (Zhu et al., 2018). Experiments have shown that the combination of Realgar and *S. miltiorrhiza* can effectively alleviate adverse reactions caused by Realgar, such as those involving the heart and liver (Wang et al., 2008). Sun (Sun, 2020) found that Realgar affects the IF composition of normal mice in a dose-dependent manner, reduces the abundance of *Firmicutes* and *Bacteroidetes*, and increases the abundance of *Proteobacteria*. Disturbances in the IF make Realgar more toxic and cause higher mortality. After transplanting normal mouse flora into a model of flora disorder, the toxicity of Realgar could be alleviated by improving the IF disorder. This indicates that the IF is a key factor in the toxicity of Realgar. In an acute promyelocytic leukemia (APL) mouse model, Realgar increased the intestinal permeability of APL mice. When combined with *S. miltiorrhiza*, *S. miltiorrhiza* can reversed the adverse effects of Realgar. *Akkermansia_muciniphila* is a specific flora regulated by *S. miltiorrhiza* combined with Realgar, and is related to the occurrence of colitis. Therefore, Realgar can disturb the IF of APL mice and improve intestinal permeability in APL mice. *S. miltiorrhiza* reduces intestinal permeability and alleviates Realgar toxicity by repairing the intestinal mucosal barrier, which may be associated with IF.

Moreover, many studies have investigated the relationship between the attenuation effect of TCMC and the IF, as shown in Table 2.

**TABLE 2:** Some research about the relationship between the attenuation effect of TCMC and IF.

Drug pair	Model	Flora change	Action mechanism of drug pair
*Panax ginseng* C. A. Mey. [Araliaceae; Ginseng radix et rhizome rubra] (*P. ginseng*) and *Aconitum carmichaelii* Debeaux [Ranunculaceae; Aconite lateralis radix praeparata] (*A. carmichaelii*) Tang et al. (2018)	Normal rats	The results of flora analysis showed that the abundance of beneficial bacteria *Lactobacillus* and *Bacteroides* in the 1:2 extract group was higher than that in the ethanol extract and 1:1 extract groups	Different proportions of *P. ginseng* and *A. carmichaelii* have different effects on the IF of rats. The increase in the proportion of *P. ginseng* can improve the intestinal microecological environment and promote the growth of beneficial bacteria to a certain extent
Z. jujuba and *Croton tiglium* L. [Euphorbiaceae; Crotonic semen pulveratum] (*C. tiglium*)Li et al. (2019a)	Normal mice	Compared with the *C. tiglium* group, *C. tiglium* combined with Z. jujuba can effectively reduce the relative abundance of inflammatory-related bacteria *Sphingomonas*, *Oscillospira*, and *Bilophila* and can specifically regulate the abundance of *Lactococcus*	The compatibility of Z. jujuba and *C. tiglium* can show a certain trend in the aspects of serum immune indicators, intestinal movement, intestinal injury, and IF structure
*Panax notoginseng* (Burkill) F. H. Chen ex C. H. Chow [Euphorbiaceae; Notoginseng radix et rhizoma] (*P. notoginseng*) and *Periploca sepium* Bunge [Apocynaceae; Periploca cortex] (*P. sepium*) Li L. et al. (2019)	Normal rats	After the combination of periplocin and *P. notoginseng* saponins, there was no significant difference in the diversity of the flora, but the relative abundance of *Bacteroides* increased significantly, while the relative abundance of *Lactobacillus* decreased	The increase in the number of total bacteria and dominant bacteria in the combination group of periplocin and *P. notoginseng* saponins reflects the detoxification effect of *P. notoginseng* saponins and preliminarily reveals the mechanism of the combination of the two drugs from the perspective of regulating IF
*C. chinensis* and *Radix Aucklandiae* [Asteraceae; Aucklandiae radix] (*R. Aucklandiae*) Wang T. et al. (2022)	Normal mice	Compared with the normal group, the ethanol extract of *R. Aucklandiae* has less impact on the IF. *C. chinensis* alkaloids reduce the diversity of IF, while the combination of different doses of drugs significantly increases the diversity and dose-dependently increases the abundance of *Rikenellacae RC9* and *Lactobacillus* and reduces the abundance of *Psychrobacter, Bacteroides,* and *Ruminococcus*	The ethanol extract of *R. Aucklandiae* alleviates the adverse reactions caused by *C. chinensis* alkaloids by regulating gastrointestinal function, intestinal microbiota composition, and metabolic disorders (Figure 5)

**FIGURE 5:**
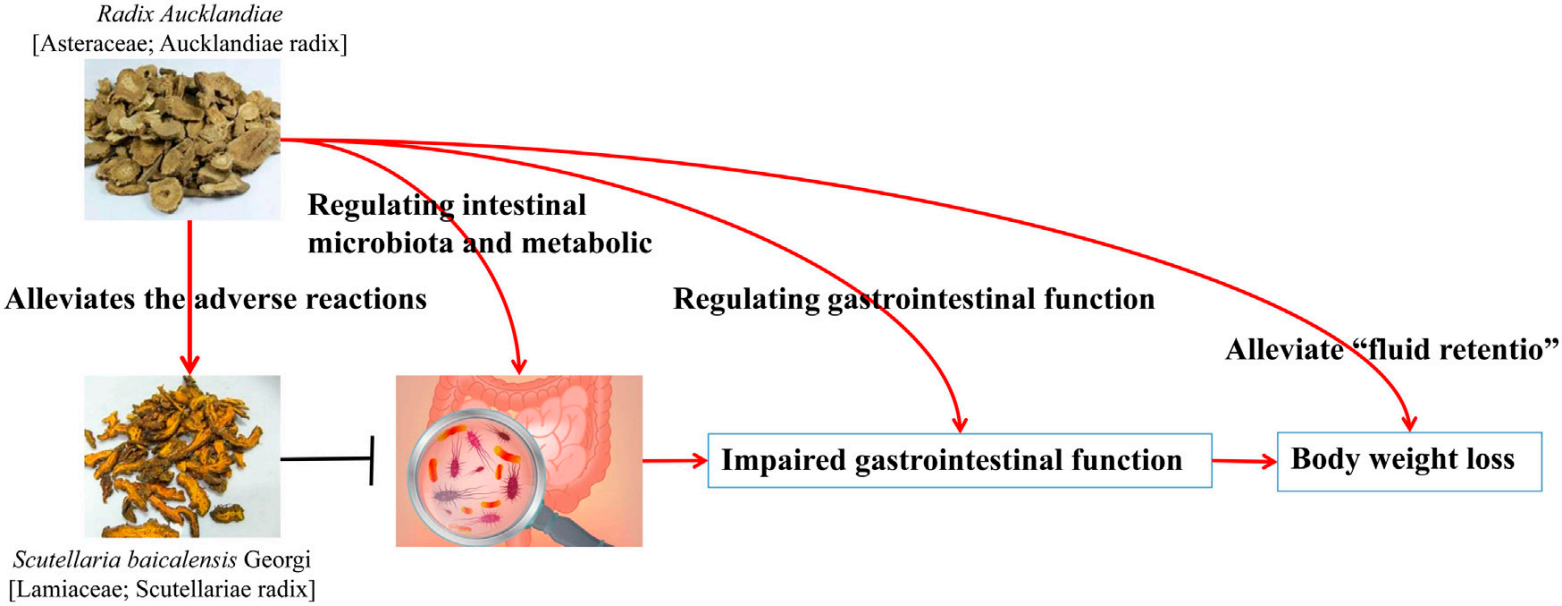
Adverse reactions to the ethanol extract of *R. Aucklandiae* caused by Coptidis alkaloids by regulating the composition of intestinal microflora (Wang T. et al., 2022).

## 4 Current situation of research on the relationship between the incompatibility effect of CM and IF

“Xiang Wu” and “Xiang Fan” are both contraindicated combination of TCM. “Xiang Wu” refers to one drug acting in combination with another, resulting in reduced or even loss of efficacy. For example, the effect of *P. ginseng* on promoting energy metabolism, regulating immune and antioxidation in the spleen qi deficiency rats were decreased after the compatibility of *P. ginseng* and *V. nigrum* L (Melanthiaceae; Veratrum nigrum) (*Veratrum nigrum*) (Lin H. et al., 2016). “Xiang Fan” refers to the occurrence of severe toxic reactions or side effects when two drugs are combined. Chen (Chen Y.Y. et al., 2019) conducted a contraindication evaluation on the compatibility of *D. genkwa* Siebold & Zucc (Thymelaeaceae; Genk flos) (*Daphne genkwa*) and *G. uralensis*, and found that the combination of *D. genkwa* s and *G. uralensis* showed severe liver, kidney, and reproductive organ toxicity in rats. *Euphorbia kansui* T. N. Liou ex S. B. Ho (Euphorbiaceae; Kansui radix) (*E. kansui*) alone has no obvious toxicity, but it can show toxicity when combined with *G. uralensis*, and the toxicity increases with the increase of the proportion of *G. uralensis* (Juan et al., 2015). The “Shiba Fan” obtained by summarizing the rule is one of the most representative theories of TCM contraindicated combination. Although the “Shiba Fan” of TCM has existed for millennia, and there are many studies about the mechanism in recent decades, and even the Pharmacopoeia stipulates that “Shiba Fan” cannot be used together in the form of law, the specific mechanism of “Fan” has not yet been proved. After summarizing previous studies, we believe that “Xiang Wu” or “Xiang Fan” of the two drugs is also related to the regulation of IF. We speculated that a drug plays a better therapeutic role by increasing the abundance of beneficial bacteria and decreasing the abundance of harmful bacteria. However, when combined with another drug, the structure of the IF changes, resulting in reduced efficacy or even loss of efficacy. This may be a possible mechanism for the effect of “Xiang Wu.” The possible mechanism of “Xiang Fan” may be that the compatibility of the two drugs specifically increases the abundance of harmful bacteria and decreases the abundance of beneficial bacteria, so it manifests as a toxic reaction or side effect.

**FIGURE 6:**
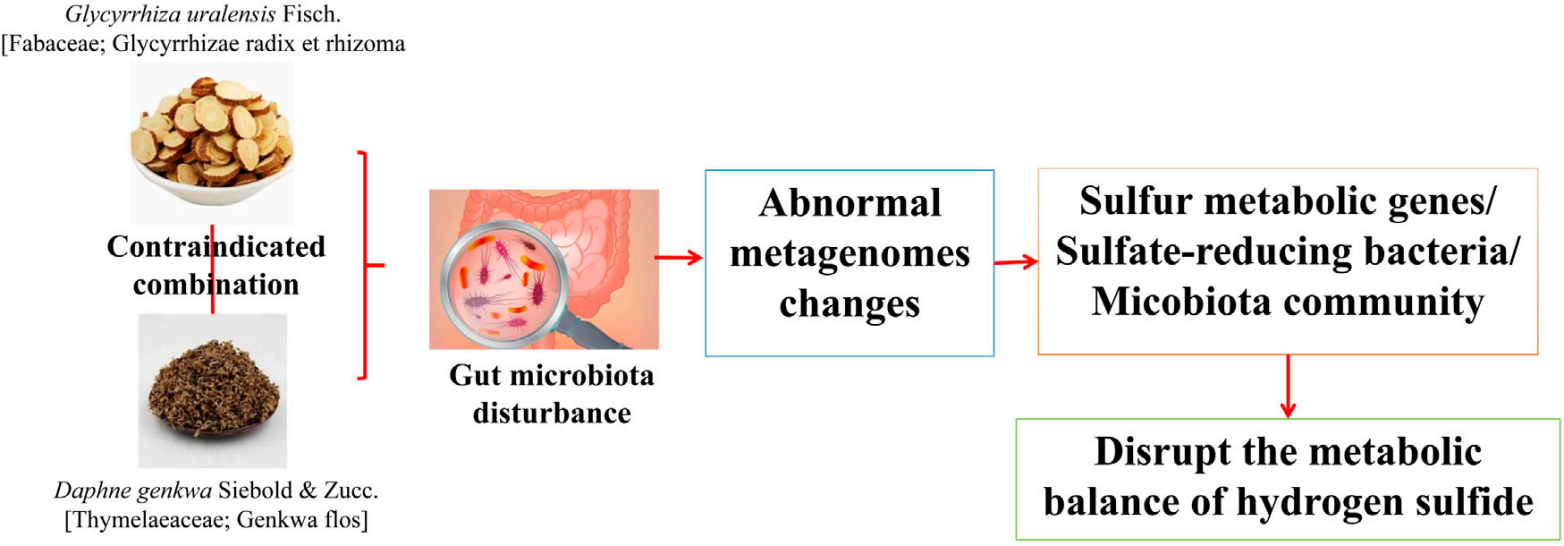
Combination of *G. uralensis* and *D. genkwa* produces toxic and side effects by affecting intestinal flora (Yu et al., 2019).

The “Fan” drug combination of *G. uralensis* and *D. genkwa* is the representative combination in the “Shiba Fan”. Yu (Yu et al., 2019) found that, compared with the use of *G. uralensis* or *D. genkwa* alone, the combination of *G. uralensis* and *D. genkwa* significantly changed the IF structure in mice. *G. uralensis* or *D. genkwa* use alone caused the abundance of 3 and 2 genera to change, respectively, whereas combined use caused the abundance of 13 genera to change significantly. Among them, the combination of *G. uralensis* or *D. genkwa* specifically increased the abundance of *Bacillus* and increased the abundance of *Desulfovibrio*, which produced H_2_S nine times, indicating that the combination of *G. uralensis* and *D. genkwa* greatly enhanced their ability to regulate the IF community structure. Macrogenomic prediction analysis showed that hydrogen sulfide metabolism-related genes appeared in the first 20 differential chemical reactions caused by *G. uralensis* or *D. genkwa*, and the abundance of these 10 genes further increased in the combined *G. uralensis* and *D. genkwa* group. Moreover, through the detection of hydrogen sulfide levels in mouse feces and serum, it was found that the combination of *G. uralensis* and *D. genkwa* significantly increased the content of hydrogen sulfide in mouse feces and significantly reduced the concentration of hydrogen sulfide in mouse serum, indicating that the combination of *G. uralensis* and *D. genkwa* could disrupt the metabolic balance of hydrogen sulfide in the mouse intestine. The combination of *G. uralensis* and *D. genkwa* showed obvious negative effects in regulating the IF community structure and hydrogen sulfide metabolism, which may be related to “increasing toxicity” (Figure 6).

Tao (Tao et al., 2018) studied the toxicity and side effects of a combination of *Euphorbia lathyris* L (Euphorbiaceae; Euphorbia Semen) (*E. lathyris*) and *G. uralensis* in normal mice and found that *G. uralensis* had no significant impact on the gastrointestinal tract. *E. lathyris* damages the intestinal mucosa, thus damaging the intestinal barrier function and weakening gastrointestinal motility regulation. The combination of *G. uralensis* and *E. lathyris* significantly enhanced *E. lathyris* damage to the intestinal mucosa. The results of the intestinal microbial analysis showed that *G. uralensis*, *E. lathyris*, and their combination caused changes in the IF structure. The levels of beneficial bacteria, *Lactobacillus*, were significantly reduced after *E. lathyris* administration, reflecting the intestinal toxicity of *E. lathyris*. The characteristic differences caused by the combination of *G. uralensis* and *E. lathyris* included *Enterococcus, S24_ 7_ ukn*, *Candidatus arthromitus*, *Roseburia*, and *Erysipelotrichaceae_incertae_se-dis*. Different bacterial populations with increased abundance were associated with toxicity and side effects to varying degrees. *Enterococcus* is a common opportunistic pathogen and *S24_ 7_ Un* is one of the main lipopolysaccharide synthesizers in animal intestines. The increase in this bacterium will lead to an increase in intestinal endotoxin production, thus disrupting intestinal immune function or damaging intestinal mucosa (Kang et al., 2017); *Erysipelothrichaceae* is involved in the pathogenesis of chronic heart failure, and this flora is one of the core bacteria missing in patients with chronic heart failure (Luedde et al., 2017). According to the IF analysis, the combination of *G. uralensis* and *E. lathyris* probably aggravates intestinal injury through the abnormal regulation of the IF and its function. The results of the metagenomic analysis showed that the combination of *G. uralensis* and *E. lathyris* increased the content of genes related to aromatic amino acid degradation and mucus degradation functions, which was significantly different from the single-use group. This indicated that the combination of *G. uralensis* and *E. lathyris* changed the regulatory effect of a single drug, resulting in new and harmful regulatory effects, and then increased the production of intestinal urinary toxins and other toxic substances, causing or aggravating the risk of disease.

Further, many studies have been conducted on the relationship between the incompatibility effect of TCMC and IF, as shown in Table 3.

**TABLE 3:** Some research about the relationship between the incompatibility effect of CM and IF.

Drug pair	Model	Flora change	Action mechanism of drug pair
*P. ginseng* and *V. nigrum* Lin et al. (2022)	Ovariectomized rats	The flora structure and abundance of the model group were significantly different from those of the sham operation group. The IF structure of the rats in the *P. ginseng* group was similar to that of the sham operation group, while the IF structure of the *V. nigrum* group was closer to that of the model group. The combined drug group was located between the *P. ginseng* and *V. nigrum* groups, regulating the abundance of *Roseburia*, *Lachnospiracea_UCG_008*, Ruminococcaceae*_UCG_005*, and [*Eubacterium*]*_ ruminantium_group*	*P. ginseng* treatment can reverse the imbalance of the IF caused by the decrease of estrogen. *V. nigrum* does not affect ovariectomized rats. However, the compatibility of *P. ginseng* and *V. nigrum* may eliminate the therapeutic effect of *P. ginseng* by acting on the IF. The opposite mechanism of *P. ginseng* and *V. nigrum* may be related to the reduced effect of *P. ginseng*
*G. uralensis* and *C. tiglium* Li et al. (2019b)	Normal mice	Low-dose *C. tiglium* combined with *G. uralensis* can increase the level of harmful bacteria *Streptococcus* and Rikenellaceae*_ukn*. The pathogenic bacteria *Desulfovibrio* and *Streptococcaceae_ukn* relative abundance increased after the combination of high-dose *C. tiglium* and *G. uralensis*	The combination of *G. uralensis* and *C. tiglium* will affect the diuretic effect of *C. tiglium*, and the effect of the two drugs on the IF structure confirms that the combination of *G. uralensis* and *C. tiglium* has a trend of reducing efficacy and increasing toxicity
*G. uralensis* and *Euphorbia pekinensis* Rupr. [Euphorbiaceae; Euphorbia pekinensis radix] (*E. pekinensis*) Liu et al. (2021)	Normal mice	*G. uralensis* can increase the abundance of beneficial bacteria *Lactobacillus*, while its effect is eliminated when used with *E. pekinensis*. The single use of *E. pekinensis* will reduce the abundance of *Akkermania* and *Butyricimonas*, and the combined use will increase the inhibition of beneficial bacteria. In addition, the combined use of *E. pekinensis* and *G. uralensis* significantly increased the abundance of *Streptococcus* and *Prevotella*	The Fan of *E. pekinensis* and *G. uralensis* is related to their energy metabolism functions such as inhibiting beneficial bacteria, promoting the growth of conditionally pathogenic bacteria, inhibiting butyric acid production, and weakening the tricarboxylic acid cycle of the IF (Figure 7)
*G. uralensis* and *E. kansui* Yu et al. (2018)	Normal mice	The single-use of *G. uralensis* or *E. kansui* causes changes in the abundance of 1 and 2 genera, respectively, while the combined use causes significant changes in the abundance of 7 genera, with a significant reduction in *Prevotelaceae*-related genera, a 10-fold increase in the abundance of *Desulfovibrio*, which produces H_2_S, and a specific increase in the abundance of *Mycoplasma*	The combination of *G. uralensis* and *E. kansui* damages the IF community structure and its related lipid and hydrogen sulfide metabolism balance, which may pose a threat to human health (Figure 8)
*G. uralensis* and *Sargassum fusiforme* (Harv.)Setch. [Sargassaceae; Sargassum] (*S. fusiforme*) Yu, (2018)	Normal mice	*G. uralensis* increases the abundance of *Reburial* and decreases the abundance of *Mucispirillum*. *S. fusiforme* does not affect the abundance of flora, while the combination of *G. uralensis* increases the abundance of *Mycoplasma* and decreased *Mucispirillum* and *RC9_gut_group*	The combination of *G. uralensis* and *S. fusiforme* plays an adverse role in the body by regulating the IF to disrupt fructose metabolism, fatty acid metabolism, and selenium compounds metabolism

**FIGURE 7:**
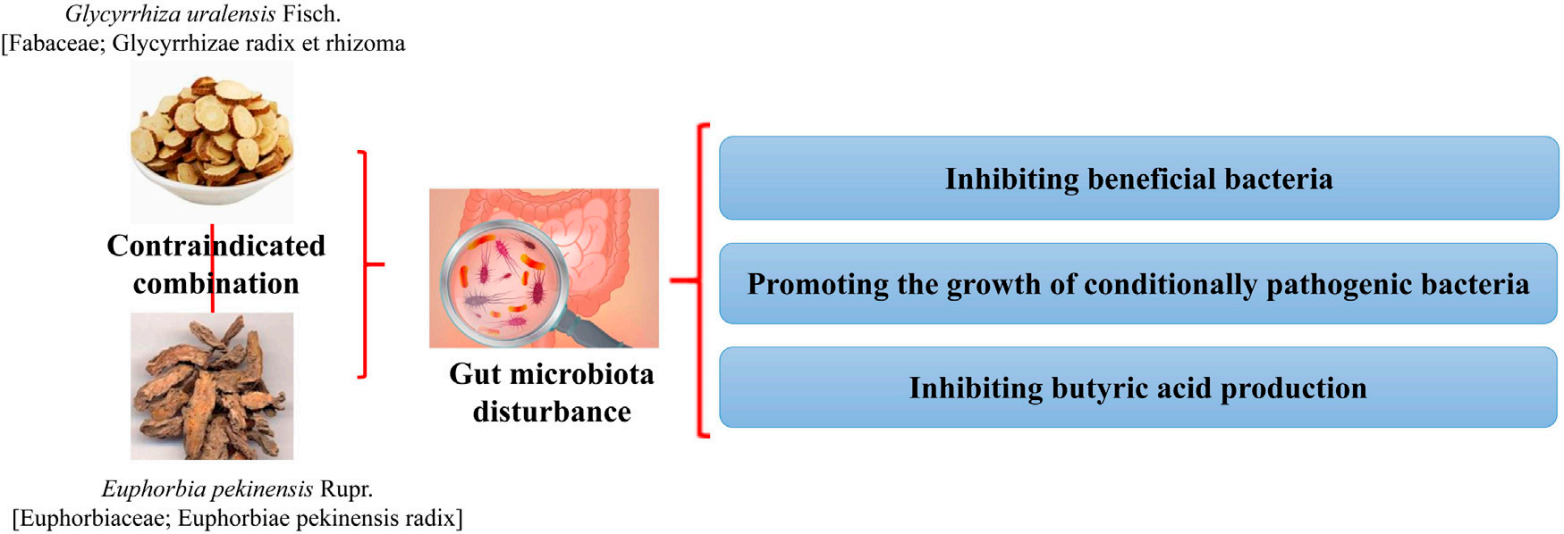
Combination of G. uralensis and *E. pekinensis* produces toxic and side effects by inhibiting beneficial bacteria and promoting the growth of conditional pathogenic bacteria (Liu et al., 2021).

**FIGURE 8:**
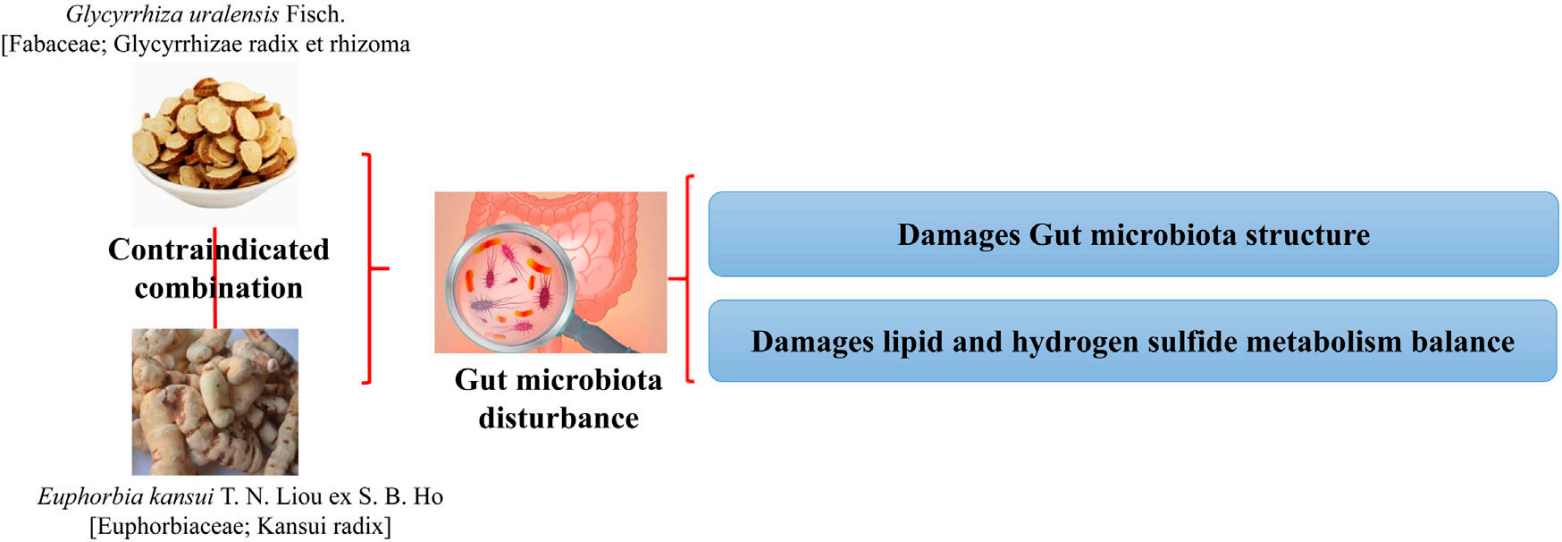
Combination of G. uralensis and E. kansui produces toxic and side effects by disrupting the structure of the intestinal flora and the associated metabolic balance (Yu et al., 2018).

## 5 Relationship between CM, the IF, and the Metabolites of the IF

CM can regulate the abundance of beneficial and harmful bacteria in the IF. For example, polysaccharides are a high proportion of components in CM, which can not only change the growth environment of the IF but can also be used as a substrate by beneficial bacteria to promote their growth of beneficial bacteria (Li et al., 2017). The organic acids, in the effect as pH buffers, can maintain the stability of the intestinal pH and provide a suitable environment for the proliferation of beneficial bacteria. In addition, the metabolites produced by beneficial bacteria can indirectly inhibit the growth of harmful bacteria. Some CM can directly inhibit the growth of pathogenic microorganisms, thereby regulating the intestinal microecological balance. Heat-clearing CM has a significant inhibitory effect on harmful bacteria (Xiao et al., 2019). Toxic CM, such as *Tripterygium wilfordii*, can effectively reduce the number of harmful bacteria, including *Enterobacteriaceae, Enterococcus,* and *Bacteroides*, in the intestines of UC mice and rats with IgA nephropathy (Ren et al., 2020; Wu et al., 2020). Therefore, CM can alter the metabolic products of the IF by adjusting the overall structure of the flora.

As a bridge between the IF and the body, the metabolites of the IF are mainly SCFAs. SCFAs are composed of 1–6 carbon atoms and are products of fermentation by IF. The SCFAs include acetic, propionic, and butyric acids. The production and consumption of SCFAs are dynamic processes, and their content reflects the activity of bacteria and the number of bacterial populations. SCFAs also affect energy metabolism, mucosal growth, and cell differentiation. SCFAs are not only anti-inflammatory but also reduce the pH in the intestine to inhibit harmful bacteria and balance the IF, and can maintain the balance of water and electrolytes and stimulate the secretion of hormones in the gastrointestinal tract. Therefore, SCFAs are closely associated with many diseases, including ulcerative colitis, obesity, diabetes, nonalcoholic fatty liver disease, autism, airway allergic inflammation, and hypertension (Shao et al., 2019). The IF is also involved in BA metabolism. In the liver, cholesterol is converted to primary free Bas through a multistage enzymatic reaction. Primary free Bas bind to taurine and glycine in the liver, convert them into conjugated Bas, and pass them through the biliary tract for discharge into the intestinal tract. Under the conjugate action of IF, taurine or glycine is removed, and the conjugated Bas become secondary Bas. Secondary Bas return to the liver through the portal system to continue binding. This is known as the enterohepatic cycle. Various Bas form Bas pools in different proportions and act on the host through Bas receptors such as the farcesoid X receptor and G-protein-coupled bile acid receptor, thereby affecting host metabolism, glycolipid metabolism, and energy homeostasis (Guo et al., 2022).

## 6 Problems and suggestions of the study on the connotation of the IF and CM

The occurrence and development of diseases and the efficacy of CM are closely related to IF. In summary, we found that the effect of a single drug on the regulation of the IF was different from its compatibility. The composition of the IF regulated by CM combinations is not a simple superposition of the effects of two individual drugs; the compatibility of drugs also plays a specific role in regulating intestinal metabolites, thus producing a different pharmacodynamic effect. This may be the angle from which the compatibility mechanism can be clarified. At present, the research on intestinal microbiota in TCM is still in its infancy. By summarizing previous research results, we provide suggestions for research on intestinal microbiota in terms of compatibility.

First, when studying the relationship between compatibility and IF, most of the research objects are drug pairs or whole prescriptions, but did not involve the comparison of changes in the IF before and after the treatments. Such a line of research cannot show that the changes in efficacy produced by the combination are related to the IF and cannot reflect where the characteristics of the combination lie. Therefore, we suggest that when studying the relationship between compatibility and flora, drug pairs or groupings should be studied by splitting the prescriptions. By comparing the composition and abundance changes of IF, we can find the specific flora regulated by the drug pair, and on this basis, we can further analyze the role played by the IF in the treatment of diseases by the drug pair.

Second, some studies only observed changes in the IF after drug compatibility treatment, which only showed a correlation between the compatibility of drugs, flora, and diseases, lacking verification of the causal relationship, and were unable to draw the conclusion that drugs play a therapeutic role through the action of flora, which is relatively less reliable. Therefore, we suggest that pseudo-sterile animal models of broad-spectrum antibiotic interference and fecal transplants can be used to study the role of the intestinal flora in the efficacy of drug pairs.

Third, 16s rDNA gene sequencing technology is the most widely used in IF research at present. Although this method overcomes the limitations of traditional culture methods and can provide relative abundance from the phylum to the genus level, this sequencing technology cannot identify specific changes in the IF at the species level; therefore, it is unable to identify the strains and related metabolites specifically regulated by the compatibility of drugs. It was impossible to further verify the relationship between the flora and compatibility. Therefore, we suggest the use of macrogenome sequencing. This method can not only clearly provide species-level composition information of IF, but also provide information on gene function, and on this basis, verify the role of flora in the body through specific flora colonization.

Therefore, when studying the relationship between the compatibility mechanism of CM and IF, we should systematically conduct in-depth research from the perspective of CM, IF, intestinal metabolite, and disease.

First, the prescription was decomposed into different parts, and an appropriate disease model was established. The effectiveness of compatibility was verified by comparing the efficacy of each drug and prescription. High-throughput sequencing technology was used to compare the composition and abundance of each drug and prescription in the IF of model animals, and specific bacteria regulated by the drug were identified. Second, the correlation between the changes in efficacy and flora specificity after compatibility was studied. Sterile or pseudosterile animals treated with antibiotics were used to observe the correlation between the IF and the occurrence and development of diseases. Flora transplantation is used to verify the therapeutic effect of specific flora on diseases and to study whether the therapeutic effect of compatible drugs can be transmitted through feces. Finally, the modes of action of the specific bacteria and their bodies were studied. However, IF may play a therapeutic role by directly acting on intestinal tissues (Mai and Draganov, 2009). In contrast, the IF affects body balance by regulating metabolites. SCFAs formed by the IF can affect energy metabolism, mucosal growth, cell differentiation, and other activities (Shao et al., 2019). Intestinal bacteria also affect Bas metabolism and regulate host metabolism, glucose metabolism, lipid metabolism, and energy homeostasis (Thomas et al., 2008). By studying the regulatory effect of compatible drugs on various metabolites after they act on IF, we can observe the influence of the drugs on the body to clarify the mechanism of drug compatibility.

## 7 Summary

The research on intestinal microorganisms is developing rapidly. Research on intestinal microorganisms provides a new perspective for us to understand the occurrence law of diseases and the mechanism of drug efficacy, as well as a new angle to clarify the theory of compatibility of CM, which is worthy of in-depth study. This paper summarizes the relationship between changes in the IF and its metabolites after compatibility with CM and the synergism, toxicity reduction, and toxicity enhancement after compatibility with CM. These studies show that the special effects of CM compatibility are related to the specific regulation of the IF and its metabolites by the drug; however, the current research still has some limitations. Therefore, this study suggests conducting in-depth research from the perspective of drug pair prescription–IF–intestinal metabolite–organism–disease to provide help for scientific research on the compatibility mechanism of CM in the future.

## Figures and Tables

**Table:** 

**CM**	Chinese medicines
**TCM**	traditional Chinese medicine
**TCMC**	traditional Chinese medicine compatibility
**IF**	intestinal flora
**BAs**	bile acid
**SCFAs**	short-chain fatty acid
***S. baicalensis***	*Scutellaria baicalensis* Georgi (Lamiaceae; Scutellaria radix)
***C. chinensi*** *s*	*Coptis chinensis* Franch. (Ranunculaceae; Coptidis Rhizoma)
**UC**	ulcerative colitis
**T2DM**	type 2 diabetes mellitus
**DSS**	dextran sulfate sodium
***S. japonica***	*Sophora japonica* Linn (Fabaceae; Sophora Flos)
**F/B**	*Firmicutes*/*Bacteroidetes*
**GQD**	Geogen Qilian Decoction
**AE**	acute enteritis
***P. montana***	*Pueraria montana* var. *Lobata* (Willdenow) Maesen and S. M. Almeida ex Sanjappa & Predeep (Fabaceae; Pueraria Lobata Radix)
***G. uralensis***	*Glycyrrhiza uralensis* Fisch. (Fabaceae; Glycyrrhizae radix et rhizoma)
**BXD**	Bangia Xiexin Decoction
***P. ternata***	*Pinellia ternata* (Thunb.) Ten. ex Breitenb. (Araceae; Pinelliae rhizoma)
***Z. officinale***	*Zingiber officinale* Roscoe (Zingiberaceae; Zingiberis rhizoma)
***P. ginseng***	*Panax ginseng* C. A. Mey. (Araliaceae; Ginseng radix et rhizoma)
***Z. jujuba***	*Ziziphus jujuba* Mill. (Rhamnaceae; Jujube fructus)
***G. max***	*Glycine* max (Linn.) Merr. (Fabaceae; Sojae Semen Praeparatum)
***G. jasminoides***	*Gardenia jasminoides* J. Ellis (Rubiaceae; Gardenia fructus)
**ZCD**	Zizia Chi Decoction
***S. miltiorrhiza***	*Salvia miltiorrhiza* Bunge (Lamiaceae; Salvias miltiorrhizae radix et rhizoma)
**APL**	acute promyelocytic leukemia mice model
***V. nigrum***	*Veratrum nigrum* L. (Melanthiaceae; Veratrum nigrum)
***E. kansui***	*Euphorbia kansui* T. N. Liou ex S. B. Ho (Euphorbiaceae; Kansui radix)
***D. genkwa***	*Daphne genkwa* Siebold and Zucc. (Thymelaeaceae; Genk flos)
***E. lathyris***	*Euphorbia lathyris* L. (Euphorbiaceae; Euphorbia Semen)
***A. caruifolia***	*Artemisia caruifolia* Buch.-Ham. ex Roxb. (Asteraceae; Artemisiae annuae herba)
***P. sinensis***	*Pelodiscus sinensis* (Trionychidae; Trionycis carapace)
***R. palmatum***	*Rheum palmatum* L. (Polygonaceae; Rhein radix et rhizoma)
***A. membranaceus***	*Astragalus membranaceus* var. *mongholicus* (Bunge) P.K.Hsiao (Fabaceae; Astragali radix)
***A. macrocephala***	*Atractylodes macrocephala* Koidz. (Asteraceae; Atractylodis Macrocephalic rhizoma)
***C. pilosula***	*Codonopsis pilosula* (Franch.) Nannf. (Campanulaceae; Codonopsis radix)
***R. glutinosa***	*Rehmannia glutinosa* (Gaert.) Libosch. ex Fisch. et Mey. (Orobanchaceae; Rehmanniae radix)
**BHD**	Baidu Decoction
**G. Fibrous**	Gypsum Fibrous
***A. asphodeloides***	*Anemarrhena asphodeloides* Bunge (Asparagaceae; Anemarrhenae rhizoma)
**SP**	Swishen Pill
**QHTF**	Quyu Huatan Tongmai Fang
**LPY**	LianPu Yin
***H. officinalis***	*Houpoea officinalis* (Rehder and E. H. Wilson) N. H. Xia and C. Y. Wu (Magnoliaceae; Magnolia officinalis cortex)
***A. carmichaelii***	*Aconitum carmichaelii* Debeaux (Ranunculaceae; Aconite lateralis radix praeparata)
***C. tiglium***	*Croton tiglium* L. (Euphorbiaceae; Crotonic semen pulveratum)
***P. notoginseng***	*Panax notoginseng* (Burkill) F. H. Chen ex C. H. Chow (Euphorbiaceae; Notoginseng radix et rhizoma)
***P. sepium***	*Periploca sepium* Bunge (Apocynaceae; Periploca cortex)
***R. Aucklandiae***	*Radix Aucklandiae* (Asteraceae; Aucklandiae radix)
***E. pekinensis***	*Euphorbia pekinensis* Rupr. (Euphorbiaceae; Euphorbia pekinensis radix)
***S. fusiforme***	*Sargassum fusiforme* (Harv.) Setch. (Sargassaceae; Sargassum)

## References

[B1] Beerens H. Romond C. (1977). Sulfate-reducing anaerobic bacteria in human feces. Am. J. Clin. Nutr. 30 (11), 1770–1776. 10.1093/ajcn/30.11.1770 920636

[B2] Borton M. A. Sabag-Daigle A. Wu J. Solden L. M. O'Banion B. S. Daly R. A. (2017). Chemical and pathogen-induced inflammation disrupt the murine intestinal microbiome. Microbiome 5 (1), 47. 10.1186/s40168-017-0264-8 28449706 PMC5408407

[B3] Chen J. Zhang L. K. Gu W. C. Xin sheng Z. Ling L. Tao H. (2021). Effect of Bangia Xiexin Decoction on intestinal flora of mice with ulcerative colitis induced by dextran sodium sulfate. China J. Chin. Materia Medica 46 (11), 2871–2880. 10.19540/j.cnki.cjcmm.20210119.401 34296588

[B4] Chen L. Zhang L. Guan X. l. Fang Z. R. Wang W. M. (2019). The effect of Zizia-Chi decoction and its decomposed recipe on six gut bacteria. Chin. J. Microecology 31 (1), 8–11+16. 10.13381/j.cnki.cjm.201901002

[B5] Chen Y. Lu J. Zhu S. M. Wang T. T. Fan Y. H. Ji W. l. (2020). Effect of Geogen Qilian Decoction and it's different compatibility groups on gut microbiota in rats with acute enteritis based on high-throughput sequencing. China J. Chin. Materia Medica 45 (06), 1406–1417. 10.19540/j.cnki.cjcmm.20191112.401 32281355

[B6] Chen Y. Y. Tang Y. P. Shang E. X. Zhu Z. H. Tao W. W. Yu J. G. (2019). Incompatibility assessment of Genkwa Flos and Glycyrrhizae Radix et Rhizoma with biochemical, histopathological and metabonomic approach. J. Ethnopharmacol. 229, 222–232. 10.1016/j.jep.2018.10.014 30339979

[B7] Cui X. Qian D. W. Jiang S. Shang E. X. Zhu Z. H. Duan J. A. (2018). Scutellaria radix and coptidis rhizoma improve glucose and lipid metabolism in T2DM rats via regulation of the metabolic profiling and MAPK/PI3K/akt signaling pathway. Int. J. Mol. Sci. 19 (11), 3634. 10.3390/ijms19113634 30453687 PMC6274950

[B8] Dai L. Chen Q. m. Liu X. p. Lin Y. y. Li K. Yue J. (2022). Bangia xiexintang and its disassembled prescriptions regulate colonic mucosal immunity in young rats with flora disorder. Chin. J. Exp. Traditional Med. Formulae 28 (11), 42–50. 10.13422/j.cnki.syfjx.20221038

[B9] Ding M. Dong H. l. Jiang G. r. Yan S. Zong Y. (2019). Overview of research on the compatibility of scutellaria baicalensis and coptis chinensis in the treatment of type 2 diabetes. China Pharm. 30 (17), 2440–2444. 10.6039/j.issn.1001-0408.2019.17.27

[B10] El-Baz A. M. Khodir A. E. Adel E.-S. M. M. Shata A. (2020). The protective effect of Lactobacillus versus 5-aminosalicylic acid in ulcerative colitis model by modulation of gut microbiota and Nrf2/Ho-1 pathway. Life Sci. 256, 117927. 10.1016/j.lfs.2020.117927 32526285

[B11] Fan H. Wang A. Wang Y. Sun Y. Han J. Chen W. (2019). Innate lymphoid cells: Regulators of gut barrier function and immune homeostasis. J. Immunol. Res. 2019, 2525984. 10.1155/2019/2525984 31930146 PMC6942837

[B12] Guan Y. Chen K. Quan D. Kang L. Yang D. Wu H. (2021). The combination of scutellaria baicalensis Georgi and Sophora japonica L. Ameliorate renal function by regulating gut microbiota in spontaneously hypertensive rats. Front. Pharmacol. 11, 575294. 10.3389/fphar.2020.575294 33643031 PMC7907655

[B13] Guo X. Okpara E. S. Hu W. Yan C. Wang Y. Liang Q. (2022). Interactive relationships between intestinal flora and bile acids. Int. J. Mol. Sci. 23 (15), 8343. 10.3390/ijms23158343 35955473 PMC9368770

[B14] Han Y. Li C. N. Huan Y. Sun S. j. Mu Y. z. Shen Z. f. (2016). Effects of berberine compatible with stachyose on glycolipid metabolism and gut microbiota in diabetic mice. Chin. J. Clin. Pharmacol. 32 (12), 1121–1124. 10.13699/j.cnki.1001-6821.2016.12.021

[B15] Juan S. Yu-ping T. Shu-jiao L. Wei-wei T. Shu-an S. Da-wei Q. (2015). Factor analysis on the dosage-toxicity relationship of kansui-licorice. China J. Traditional Chin. Med. Pharm. 30, 1531–1537.

[B16] Kang C. Wang B. Kaliannan K. Wang X. Lang H. Hui S. (2017). Erratum for Kang et al., "Gut Microbiota Mediates the Protective Effects of Dietary Capsaicin against Chronic Low-Grade Inflammation and Associated Obesity Induced by High-Fat Diet". mBio 8 (3), e00900–e00917. 10.1128/mBio.00900-17 28536285 PMC5442453

[B17] Larsen J. M. (2017). The immune response to Prevotella bacteria in chronic inflammatory disease. Immunology 151 (4), 363–374. 10.1111/imm.12760 28542929 PMC5506432

[B18] Li C. Wang Y. Y. Li J. P. Yu meng W. Sen Z. Jin'ao D. (2020). Analysis of the strategy to intervene the progress of inflammatory bowel disease by targeting intestinal bacterial respiration and energy metabolism. Acta Pharm. Sin. 55 (9), 2008–2018. 10.16438/J.0513-4870.2020-0715

[B19] Li J. Huang Z. Y. Yu T. Tao X. Y. Hu Y. M. Wang H. C. (2019). Isolation and characterization of a sequence type 25 carbapenem-resistant hypervirulent *Klebsiella pneumoniae* from the mid-south region of China. BMC Microbiol. 19 (1), 219. 10.1186/s12866-019-1593-5 31533609 PMC6749629

[B20] Li L. Ma W. J. Jia Q. Ou Yang H. Z. Chang Y. X. He J. (2019). Compatibility of periplocin and Panax notoginseng saponins on intestinal flora in SD rats by Real-time fluorescence quantitative PCR and High-throughput sequencing. Drug Eval. Res. 42 (03), 432–436. 10.7501/j.issn.1674-6376.2019.03.009

[B21] Li M.-z. Qiu F.-C. Qian C. Ruo-tong L. Yu J.-y. Sun S. (2017). Research progress of Chinese medicine polysaccharides in regulating intestinal flora. Food Nutr. China 23 (12), 13–16. 10.3969/j.issn.1006-9577.2017.12.003

[B22] Li Y. Guo S. Tao W. W. Yu J. G. Su S. l. Duan J. A. (2019a). Detoxification mechanism of Jujube Fructus and Crotonic Semen Pulveratum based on the toxicity of gastrointestinal system and diuretic effect in mice. Acta Pharm. Sin. 54 (1), 95–103.

[B23] Li Y. Guo S. Tao W. W. Yu J. G. Su S. l. Duan J. A. (2019b). Incompatibility mechanism of Crotonis Semen Pulveratum and Glycyrrhizae Radix et Rhizoma based on diuretic effect and intestinal flora structure. China J. Chin. Materia Medica 44 (3), 518–525. 10.19540/j.cnki.cjcmm.20181012.002 30989917

[B24] Liang K. Fan Y. h. Xu Y. w. Yang G. z. An R. Wang X. h. (2021). Interaction between Banxia Xiexin Decoction with its different compatibility and gastrointestinal bacteria. China J. Traditional Chin. Med. Pharm. 36 (10), 5887–5893.

[B25] Lin H. Liu Z. Liu Z. Lin Z. (2022). Incompatible effects of Panax ginseng and Veratrum nigrum on estrogen decline in rats using metabolomics and gut microbiota. J. Pharm. Biomed. Analysis 208, 114442. 10.1016/j.jpba.2021.114442 34749105

[B26] Lin H. Pi Z. F. Men L. H. Chen W. J. Liu Z. Q. Liu Z. Y. (2016). Untargeted metabonomics study of compatibility of Panax ginseng and Veratrum nigrum in spleen qi deficiency rat. Chin. J. Anal. Chem. 44 (11), 1755–1762. 10.11895/j.issn.0253-3820.160224

[B27] Lin X.-Y. Yu Y. R. Liu Q. P. He Z. X. Li H. C. Wen C. P. (2021). Regulating effect of Qinghao-Biejia on gut microbiota in the treatment of MRL/lpr mice. China J. Traditional Chin. Med. Pharm. 36 (11), 6743–6746.

[B28] Lin Z. Zu X. p. Xie H. s. Jin H. z. Yang N. Liu X. r. (2016). Research progress in mechanism of intestinal microorganisms in human diseases. Acta Pharm. Sin. 51 (6), 843–852. 10.16438/j.0513-4870.2015-0803 29878736

[B29] Liu C. S. Liang X. Wei X. H. Jin Z. Chen F. L. Tang Q. F. (2019). Geogen qinlian decoction treats diarrhea in piglets by modulating gut microbiota and short-chain fatty acids. Front. Microbiol. 10, 825. 10.3389/fmicb.2019.00825 31057525 PMC6482297

[B30] Liu D. Zhao R. Wu Y. Wang Y. Yang R. Ke X. (2022). Variation in the efficacy of anti-ulcerative colitis treatments reveals the conflict between precipitating compatibility of traditional Chinese medicine and modern technology: A case of scutellaria-coptis. Front. Pharmacol. 13, 819851. 10.3389/fphar.2022.819851 35517805 PMC9065555

[B31] Liu J.-x. (2019). “Study on the compatibility of Swishen Wan which is based on the pharmacodynamics of diarrhea-predominant irritable bowel syndrome,”. Master (Guiyang, China: Guizhou University).

[B32] Liu S. Qiao S. Wang S. Tao Z. Wang J. Tao J. (2021). Intestinal bacteria are involved in radix Glycyrrhizae and radix Euphorbia pekinensis incompatibility. J. Ethnopharmacol. 273, 113839. 10.1016/j.jep.2021.113839 33476713

[B33] Luedde M. Winkler T. Heinsen F. A. Ruhlemann M. C. Spehlmann M. E. Bajrovic A. (2017). Heart failure is associated with depletion of core intestinal microbiota. Esc. Heart Fail 4 (3), 282–290. 10.1002/ehf2.12155 28772054 PMC5542738

[B34] Luo Y. Zhang X. Zhang W. Yang Q. You W. Wen J. (2021). Compatibility with Semen Sojae Praeparatum attenuates hepatotoxicity of Gardenia Fructus by regulating the microbiota, promoting butyrate production and activating antioxidant response. Phytomedicine 90, 153656. 10.1016/j.phymed.2021.153656 34332844

[B35] Mai V. Draganov P. V. (2009). Recent advances and remaining gaps in our knowledge of associations between gut microbiota and human health. World J. Gastroenterol. 15 (1), 81–85. 10.3748/wjg.15.81 19115471 PMC2653298

[B36] Miao L. Peng Q. Sun M. Q. Zhang Y. H. Zhang Y. Ren C. Y. (2022). Regulatory effect of quyu huatan tonga prescription on intestinal microflora in golden hamster with hyperlipidemia. Chin. J. Exp. Traditional Med. Formulae 28 (1), 109–120. 10.13422/j.cnki.syfjx.20212403

[B37] Niwa T. (2013). Targeting protein-bound uremic toxins in chronic kidney disease. Expert Opin. Ther. Targets 17 (11), 1287–1301. 10.1517/14728222.2013.829456 23941498

[B38] Pluznick J. (2014). A novel SCFA receptor, the microbiota, and blood pressure regulation. Gut Microbes 5 (2), 202–207. 10.4161/gmic.27492 24429443 PMC4063845

[B39] Purushe J. Fouts D. E. Morrison M. White B. A. Mackie R. I. Coutinho P. M. (2010). Comparative genome analysis of Prevotella ruminicola and Prevotella bryantii: Insights into their environmental niche. Microb. Ecol. 60 (4), 721–729. 10.1007/s00248-010-9692-8 20585943

[B40] Ren R.-Y. Han X. Song C. D. (2020). Effect of Tripterygium wilfordii multiglucoside on intestinal flora and immune function in lgA nephropathy rats based on C1GALT1/Cosmc pathway. Chin. J. Pathophysiol. 36 (11), 2050–2055. 10.3969/j.issn.1000-4718.2020.11.018

[B41] Sampson T. R. Debelius J. W. Thron T. Janssen S. Shastri G. G. Ilhan Z. E. (2016). Gut microbiota regulate motor deficits and neuroinflammation in a model of Parkinson's disease. Cell. 167 (6), 1469–1480.e12. 10.1016/j.cell.2016.11.018 27912057 PMC5718049

[B42] Shao M. Tan W. Luo H. S. (2019). Progress of short chain fatty acids in the pathogenesis of various diseases. Chin. J. Gastroenterology Hepatology 28 (8), 951–954. 10.3969/j.issn.1006-5709.2019.08.026

[B43] Shi J. Zhang Y. l. Zhang X. Li H. Q. (2012). Observation on the therapeutic effect of Zizia Chi Decoction on 44 patients with depression syndrome. Chin. J. Exp. Traditional Med. Formulae 18 (18), 316–318.

[B44] Sun Y.-t. (2020). “The study of realgar's intestinal toxicity and its mechanism of detoxicity by compatibility of Salvia miltiorrhiza,”. Master (Hefei, China: Anhui Medical University).

[B45] Tang S. Liu W. Zhao Q. Li K. Zhu J. Yao W. (2021). Combination of polysaccharides from Astragalus membranaceus and Codonopsis pilosula ameliorated mice colitis and underlying mechanisms. J. Ethnopharmacol. 264, 113280. 10.1016/j.jep.2020.113280 32822821

[B46] Tang Z. Wei J. Ou Yang H. Z. He J. (2018). Effect of aconiti lateralis radix praeparata and ginseng radix et rhizoma rubra in different compounding ratio on gut microbiota in SD rats evaluated by high-throughput sequencing. Drug Eval. Res. 41 (10), 1781–1785. 10.7501/j.issn.1674-6376.2018.10.006

[B47] Tao W. Yu J. G. Chen Y. Y. Xiao D. Guo J. M. Liu P. (2018). Incompatible mechanism of compatibility of Chinese medicines based on Qianjinzi and Gancao effect on intestinal flora/barrier system. China J. Chin. Materia Medica 43 (02), 369–371. 10.19540/j.cnki.cjcmm.20171027.021 29552857

[B48] Thomas C. Pellicciari R. Pruzanski M. Auwerx J. Schoonjans K. (2008). Targeting bile-acid signalling for metabolic diseases. Nat. Rev. Drug Discov. 7 (8), 678–693. 10.1038/nrd2619 18670431

[B49] Wang J. Feng W. Zhang S. Chen L. Sheng Y. Tang F. (2019). Ameliorative effect of Atractylodes macrocephala essential oil combined with Panax ginseng total saponins on 5-fluorouracil induced diarrhea is associated with gut microbial modulation. J. Ethnopharmacol. 238, 111887. 10.1016/j.jep.2019.111887 31004726

[B50] Wang L. Zhou G. B. Liu P. Song J. H. Liang Y. Yan X. J. (2008). Dissection of mechanisms of Chinese medicinal formula Realgar-Indigo naturalis as an effective treatment for promyelocytic leukemia. Proc. Natl. Acad. Sci. U. S. A. 105 (12), 4826–4831. 10.1073/pnas.0712365105 18344322 PMC2290784

[B51] Wang Q. Mao N. f. Zhang Z. g. Lu L. Yin M. z. (2022). Study on the effect of Lianpu Drink and Coptidis-Magnolia officinalis on human intestinal flora based on *in vitro* fermentation model. Lishizhen Med. Materia Medica Res. 33, 1–3. 10.3969/j.issn.1008-0805.2022.12.22

[B52] Wang T. Zhang C. Li H. Zhou R. Ye X. Yang Y. (2022). The underlying rationality of Chinese medicine herb pair Coptis chinensis and Dolomiaea souliei: From the perspective of metabolomics and intestinal function. J. Ethnopharmacol. 289, 115065. 10.1016/j.jep.2022.115065 35122977

[B53] Wei H.-L. Li J. T. Chen Z. G. Shu guang Y. (2022). Experimental study on effects of berberine combined with 6-shogaols on intestinal inflammation and flora in mice with ulcerative colitis. China J. Chin. Materia Medica 47 (16), 4418–4427. 10.19540/j.cnki.cjcmm.20220413.401 36046871

[B54] Wu H. Yu X. H. Wang H. J. Ma G. C. Zhang C. E. Ma Z. J. (2020). Effect of Tripterygium wilfordii on intestinal flora in mice with ulcerative colitis induced by dextran sodium sulfate. Chin. Traditional Herb. Drugs 51 (02), 387–396. 10.7501/j.issn.0253-2670.2020.02.015

[B55] Xiao D. Chen Y. Z. Mo X. l. (2019). Study on antibacterial activities of 4 Chinese herbal medicine commonly used in guangdong. Pharm. Today 29 (3), 166–169. 10.12048/j.issn.1674-229X.2019.03.002

[B56] Xiao S. Liu C. Chen M. Zou J. Zhang Z. Cui X. (2020). Scutellaria radix and coptidis rhizoma ameliorate glycolipid metabolism of type 2 diabetic rats by modulating gut microbiota and its metabolites. Appl. Microbiol. Biotechnol. 104 (1), 303–317. 10.1007/s00253-019-10174-w 31758238

[B57] Xie N. Cui Y. Yin Y. N. Zhao X. Yang J. W. Wang Z. G. (2011). Effects of two Lactobacillus strains on lipid metabolism and intestinal microflora in rats fed a high-cholesterol diet. BMC Complement. Altern. Med. 11, 53. 10.1186/1472-6882-11-53 21722398 PMC3144010

[B58] Xu J. Chen H. B. Li S. L. (2017). Understanding the molecular mechanisms of the interplay between herbal medicines and gut microbiota. Med. Res. Rev. 37 (5), 1140–1185. 10.1002/med.21431 28052344

[B59] Xu J. (2014). “Study on the interaction between scutellaria-coptis herb couple and intestinal bacteria,”. Master (Nanjing, China: Nanjing University Of Chinese Medicine).

[B60] Ye H.-B. (2020). “Study on material basis and mechanism of antipyretic effect of gypsum and its compatibility,”. Doctor (Changchun: Changchun University Of Chinese Medicine).

[B61] Yu J.-G. (2018). “Basic research of TCM incompatibility of "zao ji sui yuan ju zhan cao" -biological mechanisms based on gut-gut microbiota and aquaporin proteins,”. Doctor (Nanjing: Nanjing University Of Chinese Medicine).

[B62] Yu J. Guo J. Tao W. Liu P. Shang E. Zhu Z. (2018). Gancao-Gansui combination impacts gut microbiota diversity and related metabolic functions. J. Ethnopharmacol. 214, 71–82. 10.1016/j.jep.2017.11.031 29198875

[B63] Yu J. Liu Y. Guo J. Tao W. Chen Y. Fan X. (2019). Health risk of Licorice-Yuanhua combination through induction of colonic H2S metabolism. J. Ethnopharmacol. 236, 136–146. 10.1016/j.jep.2019.01.042 30851368

[B64] Zhang Y. Dai L. R. Chen Q. M. Liu X. P. Yue J. Shi L. J. (2021). Effects of banxia Xiexin decoction and its different disassembled prescriptions on colonic mucosal barrier in juvenile mice with antibiotic-induced microbiota dysbiosis. Chin. J. Inf. Traditional Chin. Med. 29, 1–7. 10.19879/j.cnki.1005-5304.202110186

[B65] Zhong Y. (2017). “The study of rhubarb, radix astragali on chronic renal failure rat intestinal flora and the influence of metabolic toxins,”. Master (Guangzhou: Guangzhou University Of Chinese Medicine).

[B66] Zhu H. H. Wu D. P. Du X. Zhang X. Liu L. Ma J. (2018). Oral arsenic plus retinoic acid versus intravenous arsenic plus retinoic acid for non-high-risk acute promyelocytic leukaemia: A non-inferiority, randomised phase 3 trial. Lancet Oncol. 19 (7), 871–879. 10.1016/S1470-2045(18)30295-X 29884593

